# 

**Published:** 1810-07

**Authors:** 


### MEDICAL

AND

### PHYSICAL

### JOURNAL.

---

CONDUCTED BY

DR. SAMUEL FOTHERGILL,

AND

WILLIAM ROYSTON, ESQ.

---

VOL. XXIV.

FROM JULY TO DECEMBER, 1810.

---

104624
34-S
5/30

*LONDON:*

PRINTED FOR RICHARD PHILLIPS, NO. 6, BRIDGE-STREET, BLACKFRIARS.

BY E. HEMSTED, GREAT NEW STREET, FETTER LANE.

### To CORRESPONDENTS.

In answer to L. E. of Godalming, on the subject of extending the practice of vaccination among the poor and indigent, by medical practitioners confining themselves to *moderate charges*, we can inform him, that there is scarcely a large town in the British dominions, in which there is not at least one establishment for the gratuitous inoculation of the Cow-Pox, where, not only the poor and indigent, but sometimes, persons in tolerably good circumstances, avail themselves of the opportunity of securing their children from the ravages of that dreadful disease the small-pox; and we have not heard of any exaction being practised by medical men, in places where no such establishments exist.

Dr. James Bradley's communication has been received. We regret its being too long for insertion in its present state; if the Doctor will permit us to make a selection from it, we shall be happy to give some of his valuable cases and remarks to our Readers in the next Number. We shall presume upon his consent, if he does not forbid us by the 10th of the Month.

